# Redox Regulation by Priming Agents Toward a Sustainable Agriculture

**DOI:** 10.1093/pcp/pcae031

**Published:** 2024-03-29

**Authors:** Durgesh Kumar Tripathi, Javaid Akhter Bhat, Chrystalla Antoniou, Nidhi Kandhol, Vijay Pratap Singh, Alisdair R Fernie, Vasileios Fotopoulos

**Affiliations:** Crop Nano Biology and Molecular Stress Physiology Lab, Amity Institute of Organic Agriculture, Amity University Uttar Pradesh, AUUP Campus Sector-125, Noida 201313, India; Zhejiang Lab, Hangzhou 310012, China; Department of Agricultural Sciences, Biotechnology and Food Science, Cyprus University of Technology, Limassol 3036, Cyprus; Crop Nano Biology and Molecular Stress Physiology Lab, Amity Institute of Organic Agriculture, Amity University Uttar Pradesh, AUUP Campus Sector-125, Noida 201313, India; Plant Physiology Laboratory, Department of Botany, C.M.P. Degree College, A Constituent Post Graduate College of University of Allahabad, Prayagraj 211002, India; Max Planck Institute of Molecular Plant Physiology, Am Mühlenberg 1, Potsdam-Golm 14476, Germany; Department of Agricultural Sciences, Biotechnology and Food Science, Cyprus University of Technology, Limassol 3036, Cyprus

**Keywords:** Biological agents, Chemical agents, Priming, Redox regulation, Sustainable agriculture

## Abstract

Plants are sessile organisms that are often subjected to a multitude of environmental stresses, with the occurrence of these events being further intensified by global climate change. Crop species therefore require specific adaptations to tolerate climatic variability for sustainable food production. Plant stress results in excess accumulation of reactive oxygen species leading to oxidative stress and loss of cellular redox balance in the plant cells. Moreover, enhancement of cellular oxidation as well as oxidative signals has been recently recognized as crucial players in plant growth regulation under stress conditions. Multiple roles of redox regulation in crop production have been well documented, and major emphasis has focused on key redox-regulated proteins and non-protein molecules, such as NAD(P)H, glutathione, peroxiredoxins, glutaredoxins, ascorbate, thioredoxins and reduced ferredoxin. These have been widely implicated in the regulation of (epi)genetic factors modulating growth and health of crop plants, with an agricultural context. In this regard, priming with the employment of chemical and biological agents has emerged as a fascinating approach to improve plant tolerance against various abiotic and biotic stressors. Priming in plants is a physiological process, where prior exposure to specific stressors induces a state of heightened alertness, enabling a more rapid and effective defense response upon subsequent encounters with similar challenges. Priming is reported to play a crucial role in the modulation of cellular redox homeostasis, maximizing crop productivity under stress conditions and thus achieving yield security. By taking this into consideration, the present review is an up-to-date critical evaluation of promising plant priming technologies and their role in the regulation of redox components toward enhanced plant adaptations to extreme unfavorable environmental conditions. The challenges and opportunities of plant priming are discussed, with an aim of encouraging future research in this field toward effective application of priming in stress management in crops including horticultural species.

## Introduction

Since the evolution of life on earth, the environmental conditions have been constantly changing, and plant life must respond to these changes in order to survive. Human activities such as industrialization and urbanization (Nagajyoti et al. 2010) and climate change have led to further environmental deterioration, thereby decreasing crop yields and leading to food shortages (Calleja-Cabrera et al. 2020, Xia et al. 2022, Alsafadi et al. 2023). Plants are exposed to different types of abiotic stresses either sequentially or simultaneously during their life cycle. Stress may also occur in combinations, for instance, heavy metals and heat (Zandalinas et al. 2021, 2023, Chowaniec et al. 2023, Goff et al. 2023, Krzyżak et al. 2023), salinity and heat (Silva et al. 2013, Martinez et al. 2018), drought and heat (Obata et al. 2015, Perdomo et al. 2017, Tricker et al. 2018, Zandalinas et al. 2018) and having more harmful effects on crops than when they occur independently. Thus, there are many studies to uncover the effect of priming in plants subjected to different combined stresses. Among the novel strategies to increase the tolerance of plants to multiple stresses, priming including nanomaterial-based approaches has emerged as a significant tool to provide resistance to crop plants against biotic and abiotic stress factors (Guzmán et al. 2022, Liu et al. 2022, Tripathi et al. 2022, Kandhol et al. 2022a, 2023, Spanos et al. 2023).

Plant priming (also referred to as sensitization or hardening) helps plants to acclimatize naturally to future stress conditions (Filippou et al. 2013; Pagano et al. 2023). Priming is considered as the mechanism by which plants can show induced resistance against stressful conditions after a conditioning treatment. This conditioning treatment leads the plant to a primed state (PS), which can be induced by treating plants with different natural or synthetic compounds, beneficial microorganisms or bearable stress exposure. In the PS, the action of the resistance is quick and efficient to counteract the stress conditions encountered by the plants (Pastor et al. 2013, Sani et al. 2013, Kandhol et al. 2022a; Pagano et al. 2023). It has been demonstrated that in comparison to unprimed plants, there is less harmful effect of stress conditions on the physiology and growth of the primed plants (Hönig et al. 2023; Pagano et al. 2023). Significant numbers of studies have shown effects of priming against different abiotic and/or biotic stresses (Conrath 2009, Martinez-Medina et al. 2016, Hossain et al. 2018). The priming process involves the collection of signaling proteins or transcription factors (TFs) in an inactive state, phosphorylation of mitogen-activated protein kinases, deposition of different cellular compounds, changes in epigenetic processes and redox status under stress conditions which enhance the defense system (Bruce et al. 2007, Pastor et al. 2014, Conrath et al. 2015, Turgut-Kara et al. 2020). Priming is reported to orchestrate the delicate balance between oxidative and reductive reactions within plant cells, which plays a crucial role in determining the overall fitness and resilience of plants in the face of environmental challenges (González-Bosch 2018, Wang et al. 2019).

Several reviews are available which broadly discuss the mechanisms involved in relevant detail (Savvides et al. 2016, Balestrini et al. 2018, Sako et al. 2020, Kerchev et al. 2020). Furthermore, novel techniques of systems biology including proteomics, transcriptomics and genomics are important to decipher the molecular network involved in plants to cope with external factors during the priming process. By examining the interconnections between priming agents and redox signaling, we aim to discern the molecular switches that drive adaptive responses and optimize resource allocation for growth and defense. This exploration is pivotal for the development of sustainable agricultural practices, as it opens avenues for enhancing crop productivity and stress tolerance in a changing climate scenario (key terminology is described in detail in **Box 1**).

Box 1: Glossary

**Chemical Priming**: The provision of natural or synthetic single molecules that efficiently activate plant responsive mechanisms and promote plant acclimation to abiotic and biotic stresses.

**Biostimulants**: A complex mixture of compounds and/or microorganisms which improves plant growth, productivity, efficiency of nutrient uptake and crop quality as well as induces plant stress tolerance responses.

**Nanomaterials**: A newly emerged category of priming employing natural or synthetic polymers and/or NPs with promising attributes, which improves plant growth and productivity and induces plant stress tolerance against adverse environmental conditions. The combined application of nanomaterials and another priming agent as a conjugated product has great potential for application in agricultural stress management practices.

**Plant priming**: It is an adaptive strategy that improves the defensive capacity of already-established plants. In plant priming, the agents are applied to the aerial and underground plant parts.

**Seed priming**: This is a pre-sowing seed treatment with a priming agent with the aim of improving seed germination, the uniformity and vigor of seedlings and enhancing plant stress tolerance. Primed seeds have a developmental advantage compared with unprimed seeds due to improved seed germination rate and seedling establishment.

**Cellular oxidation**: Loss of electrons by the atom, molecule or ion is called oxidation. In this context, cellular oxidation is defined as the loss of electron by the cell molecules such as protein, lipid or carbohydrate, which makes them highly reactive.

**Redox signaling**: Transduction signals that are coding for cellular processes and involve electron transfer reactions among free radicals or related species, redox-active metals or reductive equivalents are known as redox signaling.

**Plant stress response**: The suite of molecular and cellular processes triggered in the plant in response to abiotic and biotic stresses.

**Reactive oxygen species**: Highly reactive chemical species which contains oxygen are known as reactive oxygen species (ROS) such as O_2_^−^ and H_2_O_2_. ROS are produced as a natural byproduct in the normal oxygen metabolism, and they play an important role in cell signaling and redox homeostasis.

**Antioxidant**: Any molecule involved in the scavenging of toxic ROS accumulation and prevents the oxidative damage in cells is called antioxidant. Antioxidants include both enzymatic and non-enzymatic molecules.

**ROS-scavenging**: Removal of excess ROS by the cellular antioxidant system to prevent oxidative damage is known as ROS-scavenging.

**Cellular redox homeostasis**: Proper regulation of the formation and removal of ROS species by the cell in order to maintain an optimum ROS level in the cell environment is called cellular redox homeostasis.

**Systemic acquired acclimation**: A systemic response developed in the plant body against abiotic stimuli, such as drought, heat, salinity, light or osmotic stress.

## Priming: Different Methodologies Used in Plants

Plant priming is carried out by the exogenous application of different priming agents that come from nature and synthetic chemistry. A wide range of priming agents have been extensively used in several studies to determine their efficacy against a variety of different individually or combinational applied stresses (Savvides et al. 2016, Van Oosten et al. 2017, Alsafadi et al. 2023, Pagano et al. 2023). Experiments have been performed in many different plant species grown either under controlled conditions such as growth chamber and greenhouse or in the field (Christou et al. 2014a, Antoniou et al. 2017, 2020, Sher et al. 2017, Ertani et al. 2018, Khan et al. 2020, Singh et al. 2021, Forni and Borromeo 2023). Based on the available literature, priming agents can be classified into three main categories: (i) chemical agents, (ii) biostimulants and (iii) nanomaterials. Each category is composed of a vast range of priming agents that continuously increase since new studies focus on newly emerging agents (Sako et al. 2020).

Chemical priming is considered as one of the best-known and well-studied of the categories. Many types of chemical metabolites have been proved to enhance plant tolerance by activating important signaling processes (Antoniou et al. 2016, Savvides et al. 2016, Sako et al. 2020, Narayan et al. 2022, Brenya et al. 2023, Jeyasri et al. 2023). Chemical priming agents can be naturally occurring primary or secondary metabolites or synthetic compounds including but not limited to melatonin, reactive oxygen–nitrogen–sulfur species (RONSS donors; Sodium nitroprusside (SNP), Sodium hydrosulphide (NaHS), hybrid donor of nitric oxide and hydrogen sulfide (NOSH), and NOSH plus acetylsalicylic acid (NOSH-A)) amino acids (e.g. proline), hormones (e.g. salicylic acid [SA]), amines (e.g. polyamines, glycine betaine), carbohydrates (e.g. trehalose and raffinose), vitamins (e.g. vitamin C—ascorbate [AsC]), nutrients (e.g. KH_2_PO_4_, KNO_3_, ZnSO_4_ and CuSO_4_), fungicides (e.g. strobilurins), organic solvents (e.g. acetic acid and ethanol) and volatile organic compounds (VOCs, e.g. green leaf volatiles; linolenic and linoleic acid) (Antoniou et al. 2016, Savvides et al. 2016, Van Dingenen et al. 2017, Sequera-Mutiozabal et al. 2017, Sako et al. 2020). The mode of action of many of these chemical agents has been elucidated, and the knowledge obtained contributes to unraveling commonalities and specificities of their modus operandi.

The second category of priming agents are plant biostimulants which have recently gained a lot of attention due to beneficial effects on plant growth and production, and plant stress tolerance and crop quality (Calvo et al. 2014, Van Oosten et al. 2017, Ali et al. 2022, Benito et al. 2022, Bhupenchandra et al. 2022, Deolu‐Ajayi et al. 2022, Mandal et al. 2023). The natural character of biostimulants compared with some agents of chemical priming makes them more acceptable by farmers and consumers since they promote sustainable agriculture by minimizing accumulative contaminants and their impacts on the ecosystems (Van Oosten et al. 2017, Zulfiqar et al. 2020, Deolu‐Ajayi et al. 2022, Mandal et al. 2023). Plant biostimulants primarily include a mixture of bioactive molecules and/or microorganisms: (i) plant-based extracts, (ii) seaweed extracts, (iii) humic and fulvic acids, (iv) protein hydrolyzates, (v) inorganic salts (e.g. phosphates, Al, Co, Na, Se and Si), (vi) complex organic material obtained for agro-industrial waste products, (vi) chitosan and other biopolymers, (vii) beneficial fungi (i.e. arbuscular mycorrhizal fungi (AMF) and arbuscular mycorrhiza fungi) and (viii) beneficial bacteria (i.e. growth-promoting rhizobacteria) (Sharma et al. 2014, du Jardin 2015, Van Oosten et al. 2017, Balestrini et al. 2018, Omidbakhshfard et al. 2020, Rouphael and Colla 2020, Zulfiqar et al. 2020, Staykov et al. 2021, Dubey and Sharma 2023, Grammenou et al. 2023).

Nanotechnology is a newly emerging and novel approach in plant priming that has a great potential for application toward improved plant growth and productivity and induces plant stress tolerant against stressful environmental conditions (Ioannou et al. 2020, Prakash et al. 2022, Kandhol et al. 2022a, Sharma et al. 2023). Protective effects of nanomaterials are attributed to the small size of nanoparticles (NPs; 1–100 nm in one dimension) and to their properties including high reactivity, solubility, biochemical activity and plant cell permeability, which rely on their high surface energy and high surface-to-volume ratio (Khan et al. 2019, Ioannou et al. 2020, Joudeh and Linke 2022; Raha and Ahmaruzzaman 2022, Tripathi et al. 2023). There are three main categories of nanomaterials that are applied in agriculture: (i) inorganic (metal and metal oxide NPs; e.g. TiO_2_, gold, Au and Ag), organic (naturally produced NPs; e.g. chitosan and hydrogels) and combined NPs (clay) (He et al. 2019, Raha and Ahmaruzzaman 2022, Tripathi et al. 2023). The usage of nanomaterials in agriculture is still under optimization; nevertheless, this technology has a great potential to be used in crop stress management practices, minimizing agricultural input and environmental impact (Spanos et al. 2023, Tripathi et al. 2023).

All the aforementioned priming agents are usually applied to the plant in relatively low concentrations, through root watering, foliar spraying and seed treatment. The two former strategies are plant priming methodologies, while the latter is well-known as the seed priming—a cost-effective and eco-friendly approach to manifest stress tolerance (Wang et al. 2015a, Hönig et al. 2023, Spanos et al. 2023, Yadav et al. 2023). Seed priming is usually achieved by soaking the seeds before sowing in priming agents such as water (hydropriming), inorganic salts (halopriming), polyethylene glycol (PEG) (osmopriming), nutrients (nutripriming), hormones (hormopriming), nanomaterials and other natural or synthetic compounds (e.g. melatonin and RONSS donors) (Hasanuzzaman and Fotopoulos 2019, Kandhol et al. 2022a, Kandhol et al. 2022b; Tripathi et al. 2022, Forni and Borromeo 2023, Pagano et al. 2023. The optimum priming strategy to be employed is mostly dependent on one or more of the following factors: (i) priming agent(s), (ii) plant species, (iii) the type of stresses and (iv) timing of application (plant growth stage). Combined application of priming agents from different categories is employed to study the synergistic or additive effect on plant tolerance and growth under adverse environmental conditions (Khan et al. 2020, Sharma et al. 2022, 2023). Preliminary optimization is important for selecting the optimum mode of priming (Savvides et al. 2016). Moreover, of great interest is the duration of priming effects or the ability of primed plant to accumulate dormant signals that can serve as “memory signals”, which are activated upon exposure to a stress factor (Hilker and Schmülling 2019). Further studies are essential to decipher the underlying molecular mechanism that keeps primed plants awake and ready to respond in a future stress experience.

## Priming: Its Impact on Cellular Oxidation, Redox Imbalance and Redox/Oxidative Signaling

**Fig. 1:**
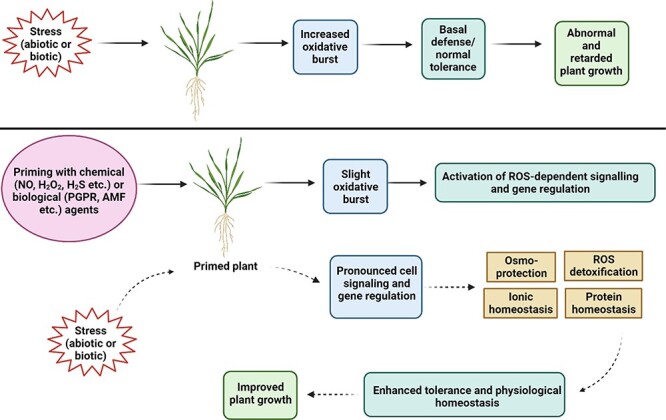
Priming-mediated impacts on plant growth via regulating redox/oxidative signaling and maintaining redox homeostasis under biotic and abiotic stresses. Created using BioRender.

Plant stresses often induce accumulation of excess ROS (e.g. H_2_O_2_), nitrogen (e.g. NO) and sulfur (e.g. H_2_S) species (collectively known as RONSS), which causes oxidative damage to cellular macromolecules (such as DNA, proteins and lipids) and cell structures, leading to permanent metabolic dysfunction resulting in the death of the plant (Anjum et al. 2015, Mandal et al. 2022). The main antioxidant enzymes involved in regulating the stress-induced oxidative damage are AsC peroxidase (APX), dehydroascorbate reductase (DHAR), monodehydroascorbate reductase (MDHAR), and glutathione (GSH) reductase (GR) (Decros et al. 2023, Mishra et al. 2023). Primed plants manage to control redox homeostasis and mitigate stress-induced cellular oxidation by regulating the production of redox cycle antioxidant molecules (GSH and ascorbic acid) and the activity of antioxidant enzymes that control the level of over-accumulated ROS (González-Bosch 2018) (**Fig. 1**). Priming modulates the oxidative environment by interacting with plant signaling pathways and promotes quick and strong responses against plant stresses. Increasing evidence has revealed that the response including physio-biochemical and molecular of primed plants against environmental stresses is regulated by oxidative signaling. Sheteiwy et al. (2016) have reported that PEG seed priming induces tolerance to Zn toxicity in rice by maintaining levels of O_2_^−^, H_2_O_2_ content and GR activity. In a recent study, soaking of wheat seed in *Moringa oleifera* leaf extract (plant-based biostimulant) and proline prominently upregulated the expression of *APX* and *GPX* (encoding for glutathione peroxidase) and increased the contents of AsC and GSH and their redox states compared with non-primed seeds under salt stress (Rady et al. 2019). In tomato seedlings, AMF inoculation is reported to induce an significant increase in GSH, AsC, redox ratio and the activity of l-galactono-1,4-lactone dehydrogenase (GalLDH) in the roots exposed to low temperature (Liu et al. 2016).

Priming with vitamins has been analyzed in several recent studies (Molnár et al. 2023, Zrig et al. 2023) such as the use of thiamine treatment, which also modulates various key regulators of NADPH oxidase (an enzyme important for H_2_O_2_ production) during plant response to pathogen stresses, as reported in *Arabidopsis* plants inoculated with *Sclerotinia sclerotiorum* (Zhou et al. 2013). Similarly, Zhang et al. (2009) have demonstrated rapid accumulation of H_2_O_2_ induced by riboflavin treatment in *Arabidopsis* plants that serve as a key element to induced resistance against *Pseudomonas syringae*. Nianiou-Obeidat et al. (2017) have also observed that riboflavin induces upregulation of glutathione-*S*-transferase belonging to phase II detoxification enzymes. Riboflavin also induces resistance against *Botrytis cinerea* and *P. viticola* in plants via its effect on H_2_O_2_ signaling (Boubakri et al. 2013, Zhou et al. 2013, Nianiou-Obeidat et al. 2017).

Similarly, phytohormone-induced priming is also a very effective and significant research area and several studies have been conducted to explore the possibilities of phyto-hormonal priming against stress conditions in plants (Rhaman et al. 2020, Liu et al. 2022, Sghayar et al. 2023). Priming with hormones can also regulate RONSS in maintaining redox homeostasis as suggested in many studies. Application of jasmonic acid (JA) in *Arabidopsis thaliana* significantly increased H_2_S content, the activities of redox cycle enzymes (GalLDH, APX, GR, MDHAR and DHAR) and the ratio of AsC/DHA (dehydroascorbic acid), while in the presence of H_2_S inhibitor (HT- hypotaurine), these effects were suppressed (Shan et al. 2018). Another study from the same group demonstrated that exogenously applied ABA in wheat seedlings regulates the AsC–GSH cycle via H_2_S production (Shan et al. 2017). Kaya et al. (2020) proposed that SA treatment enhanced AsC–GSH cycle and antioxidant defense systems in salt-stressed pepper plants via endogenous NO production. Similar findings have been demonstrated in salt-stressed *Vigna angularis* pre-treated with sole and combined application of NO and SA (Ahanger et al. 2020). Melatonin treatment in *Malus* species decreased the H_2_O_2_ content by inducing the expression of genes involved in AsC/GSH cycle (*cAPX, DHAR1, DHAR2, MDHAR* and *cGR*) under salinity and nutrient deficiency (Li et al. 2016). Remarkably, exogenous priming with redox antioxidants also alleviates oxidative stress by regulating GSH and AsC metabolism as well as the redox status and antioxidant defense system. Zhou et al. (2017) documented that exogenous application of GSH in tomato seedlings induced transcript expression levels and the activities of enzymes taking part in GSH synthesis such as GST, GPX and GR and increased the content of GSH and the ratio of GSH/GSSG. Similarly, seed priming with a mixture of AsC, proline and GSH significantly increased AsC, GSH and proline content and increased the activity of CAT (Catalase), GR and APX enzymes compared with non-primed plants under drought stress (Semida et al. 2018).

It is well-known that production of RONSS at low/optimum concentration acts as signaling molecules that orchestrate a range of molecular responses and thereby play a pivotal role in plant acclimation to stress conditions (Corpas 2017, Saddhe et al. 2019, Tomar et al. 2021). Therefore, exogenous supplementation of RONSS themselves to plants at optimum doses can also guide the plants to the PS to induce tolerance to biotic (bacteria and fungi pathogens) and abiotic (salinity, water, cold, heat and heavy metal) stresses (Antoniou et al. 2016). RONSS treatment creates minor oxidative burst that activates a redox-dependent signaling network. This in turn allows accumulation of ROS-scavenging enzymes and various TFs, and these proteins lead to the PS in plant and an enhanced stress response (Hossain et al. 2015). For example, Uchida et al. (2002) documented that H_2_O_2_ priming enhances salt tolerance in *Arabidopsis* by regulating expression of defense-related genes and similar findings are recorded in wheat seedlings (Wahid et al. 2008). Priming of cucumber plants with H_2_O_2_ induces reduction in H_2_O_2_ accumulation and mitigates the adverse effects of drought stress in H_2_O_2_-primed plants (Carvalho and Dupuy 2013), while similar findings were observed in soybean (Ishibashi et al. 2011). Three studies by Christou and colleagues demonstrated that applications of SNP (NO donor), H_2_O_2_ and NaHS (H_2_S donor) in strawberry plants result in increased production of AsC and GSH and an upregulation of their biosynthetic genes such as *GDH, GCS* and *GS*, compared with unprimed plants under salinity and heat stress conditions (Christou et al. 2013, 2014a, 2014b). Application of SNP in tomato plants ameliorated the detrimental effects of cadmium stress by enhancing the activities of redox cycle enzymes including APX, GR, MDHAR, DHAR and GST and increasing the content of AsC and GSH compared with non-primed seedlings (Ahmad et al. 2018). Similarly, a recent study by Singh et al. (2021) demonstrated that H_2_O_2_ and SNP application in soybean roots significantly induced APX, GR, MDHAR and DHAR activities and increased the GSH/GSSG and AsC/DHA redox ratios under arsenate stress.

Some priming agents also induce a wave of H_2_O_2_ in plants that transmits the local effects of a stressor to the whole plant in systemic tissues (Mittler et al. 2011, González-Bosch 2018). The H_2_O_2_ wave interacts with hormone signals and mediates the systemic acquired acclimation (SAA) in systemic tissues under stress conditions (Suzuki et al. 2013). Farnese et al. (2017) have revealed the involvement of both ROS and NO in promoting systemic acquired resistance (SAR) against external stress in plants, including stress-specific mechanisms for each type of stress. Priming with cell-wall components such as chitosan promotes H_2_O_2_ accumulation primarily through plasma membrane NADPH oxidase and induces pathogen resistance (El Hadrami et al. 2010). Fernández-Crespo et al. (2015) have documented that nutrition of tomato plants with NH_4_^+^ induces minor toxicity by inducing H_2_O_2_ accumulation, which can inturn activate SAA to increase resistance to subsequent infection of *P. syringae*.

Based on the current literature, redox homeostasis seems to be a common target for a great number of priming agents, used in several species that are exposed to a range of different stresses. Considering that maintenance of redox homeostasis is an essential element for plant acclimation to stress (Kleine et al. 2021), future research should be directed in deciphering the role of priming in the regulation of redox cycle components as a plant response to stresses.

## Priming Uses Redox Components to Regulate Genetic and Epigenetic Factors and Modulate Stress Responses

**Fig. 2:**
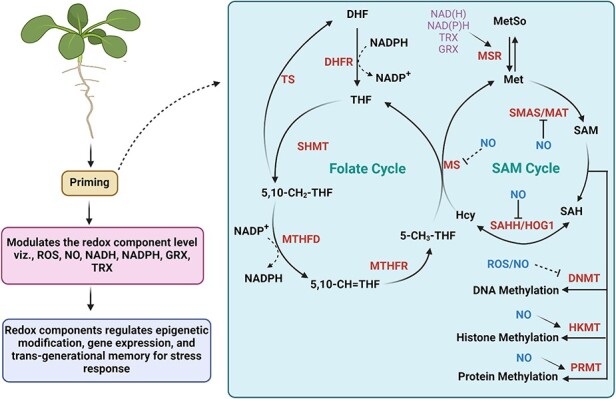
Role of priming in regulating redox components responsible for modulation of enzymes involved in genetic/epigenetic modifications—DNA methylation or histone methylation. The folate cycle initiates with the conversion of dihydrofolate (DHF) to tetrahydrofolate (THF) facilitated by DHF reductase (DHFR), using reducing equivalents from NADPH. Methyls originating from THFs (5,10-CH2-THF, 5,10-CH=THF) are produced by serine hydroxymethyl transferase (SHMT) and methenyltetrahydrofolate cyclohydrolase (MTHFD), respectively. Methylenetetrahydrofolate reductase (MTHFR) reduces 5,10-methenyl-THF to 5-methyl-THF. The methyl group from 5-methyl-THF is transferred to Hcy to synthesize Met through MS. Met generated in this process forms SAM through *S*-adenosyl methionine synthase (SAMS). SAM contributes methyl groups to DNA or proteins via DNA methyltransferase (DNMT), histone lysine methyltransferase (HKMT) or protein arginine methyltransferase (PRMT) and undergoes conversion to *S*-adenosylhomocysteine (SAH). SAH is further metabolized to Hcy through *S*-adenosylhomocysteine hydrolase (SAHH) or homologous gene silencing 1 (HOG1). Key enzymes influenced by cellular redox components include SAMS/methionine adenosyltransferase (MAT), DNMT/HKMT/PRMT, SAHH/HOG1, MS and methionine sulfoxide reductase (MSR). DHF, THF, DHFR, 5-methyl-tetrahydrofolate (5-CH3-THF), SHMT, methylenetetrahydrofolate dehydrogenase/MTHFD1, MTHFR, thymidylate synthase (TS), methionine (Met), methionine sulfoxide (MetSo), MSRs, SAM, SAHH/HOG1, SAMS/MATs, SAH, DNMT, HKMTs, PRMTs, homocysteine (Hcy) and methionine synthase (MS). Figure is adapted after modification from Saravana Kumar et al. (2020) available at CCBY4.0. Created using BioRender.

Close examination of the available literature reveals that priming agents act on redox signaling and promote faster and stronger responses to various plant stresses by regulating the oxidative environment (González-Bosch 2018, Tripathi et al. 2022, Kandhol et al. 2022a). Studies have reported transgenerational effects/memory of priming, i.e. the effect of priming can transfer to progeny of primed plants (Louis et al. 2023). For example, heat priming in wheat (Wang et al. 2016), herbivore-induced priming in *Arabidopsis* and tomato (Rasmann et al. 2012) and β-aminobutyric acid priming in common bean (Ramírez-Carrasco et al. 2017) were revealed to have a transgenerational effect. However, the genetic and epigenetic mechanisms are the most important factors regulating inter-generational/transgenerational memory (Chen and Arora 2013, Hilker et al. 2016, Avramova 2019; see **Box 2**). Redox components (oxidants and antioxidants) play an important role in regulating genetic and epigenetic perturbations in plants (Choi et al. 2007). Primary oxidants in plants include H_2_O_2_, O_2_^−^ and OH^−^, whereas antioxidants include both enzymatic components such as SOD, CAT, APX, GPX and GR and non-enzymatic ones such as NAD(P)H, NAD(H), thioredoxins (TRXs), GSH, glutaredoxin (GRX), AsC and peroxiredoxins (Hassan and Mansoor 2017, Dreyer and Dietz 2018, Laxa et al. 2019, Kumar et al. 2020). Accumulating evidence reports an important role of these redox components in regulating enzymes involved in genetic/epigenetic modifications such as DNA methylation, histone methylation and acetylation (Pétriacq et al. 2012, Xu et al. 2015a, Kumar et al. 2020, Saravana Kumar et al. 2020) and thereby modulating plant growth and vigor as well as adaptations to environmental stresses. Moreover, studies have documented priming-induced genetic/epigenetic modifications indirectly via their influence on cellular redox state which affects the transcription of defense-related genes (Espinas et al. 2016, González-Bosch 2018, Kotkar and Giri 2020, Kumar et al. 2020) (**Fig. 2**).

Box 2: Important processes involved in priming-induced epigenetic modifications

**Transgenerational memory**: Plant phenotype or stress memory that can pass from parents or even grandparents to offspring is known as transgenerational effects.

**Epigenetic modification**: Stable and heritable changes in the gene expression and function that does not involve any modification to the original sequence of DNA is termed as epigenetic modification. It includes DNA methylation and histone modifications.

**TFs**: TFs are proteins that can bind to the promoter region of the specific gene and will either turn on or turn off the transcription of the particular gene.

**RdDM**: RdDM is an epigenetic process in which double-stranded RNAs are processed to 21–24 nucleotide small interfering RNAs (siRNA) and guide methylation of homologous DNA loci.

**siRNA**: siRNA is a non-coding double-stranded RNA molecule that typically consists of 21–24 base pairs (bp), and it works in the RNA interference (RNAi) pathway.

**Glutathionylation**: It is a post-translational modification induced by the GSH in the thiol/cysteine residues of the protein. GSH is a most abundant and crucial low-molecular-mass thiol present in most cell types.

Different latest studies have shown that priming with H_2_O_2_ can enhance abiotic and biotic stress tolerance in plants by regulating detoxification of ROS as well as through regulation of different stress-responsive pathways and gene expression (Ishibashi et al. 2011, Gondim et al. 2013, Christou et al. 2014b, Gohari et al. 2020, Chattha et al. 2022, Silva et al. 2023). H_2_O_2_ priming disrupts cellular ROS homeostasis and stimulates the ROS-dependent signaling network, ROS-scavenging activity and TFs, resulting in a PS and enhanced stress responses (Hossain et al. 2015). Excess ROS levels induce DNA hypomethylation; for example, Choi et al. (2007) reported that higher levels of ROS (O^2−^) upregulate glycerophosphodiesterase-like (*NtGPDL*) by inducing demethylation in the CG sites (regions of DNA where a cytosine nucleotide is followed by a guanine nucleotide) of the *NtGPDL*-coding regions in tobacco (*Nicotiana tabacum*). Likewise, many other studies reported ROS-induced global DNA hypomethylation in various plant species including tobacco (Poborilova et al. 2015), *Pisum sativum* (Berglund et al. 2017) and *A. thaliana* (Xu et al. 2015a). Dayal et al. (2008) reported that H_2_O_2_ mediates radiation-induced transgenerational mutant phenotypes in mammalian cells, indicating a potential epigenetic effect of H_2_O_2_. The main focus of research on histone modifications in Arabidopsis plants has been their role in priming responses to abiotic stresses and biotrophic pathogens such as *P. syringae*. (Jaskiewicz et al. 2011, Luna et al. 2012). WRKY53, a transcription factor sensitive to redox changes, is triggered by H_2_O_2_ (Miao et al. 2007, Golemiec et al. 2014) in Arabidopsis plants infected with P. syringae pv. Maculicola, and this induction is associated with increased methylation of H3K4 in its promoter region. (Jaskiewicz et al. 2011). In tomato, WRKY53 was induced by the hexanoic acid- a natural priming agent (Finiti et al. 2014). ROS regulates DNA methylation by affecting the expression and activities of DNA methyl-transferases (DMTs) and DNA demethylases (DDMTs) (Xu et al. 2015a). For instance, ROS mediate DNA demethylation in *A. thaliana* by considerably downregulating domain-rearranged methyltransferase 2 expression and upregulating transcriptional abundances of DNA *Methyltransferase 1* (*MET1*) and *Demeter-like 3* (Xu et al. 2015a). Likely, NO also regulates DNA methylation by affecting DMTs; for example, NO treatment stimulates DNA hypomethylation in rice plants by downregulating DMT genes (*OsCMT2* and *OsCMT3*) and upregulating DDMT gene *OsDME* (Ou et al. 2015). Priming with low concentrations of NO resulted in enhanced heat stress tolerance in *Lablab purpureus* plants by altering DNA demethylation and methylation patterns via reduction in the levels of O_2_^.–^ and H_2_O_2_ (Rai et al. 2018).

An important role of NAD(H), NADP(H), GRXs and TRXs in the modulation of the methylation process by affecting *S*-adenosyl-l-methionine (SAM, a methyl donor for DNA and histone methylation) levels has also been decoded (Rouhier et al. 2006, Dos Santos et al. 2007, Pétriacq et al. 2012). Wang et al. (2016) reported that heat priming upregulates genes involved in the production of NADP(H), TRXs and GRX leading to transgenerational tolerance towards high temperature in wheat, suggesting epigenetic regulation. Similarly, Berglund et al. (2017) showed that treatment of pea with nicotinamide (NIC) and its natural plant metabolite nicotinic acid (NIA) prevents oxidative damage through epigenetic-mediated effects. These authors revealed that NIA and NIC priming induces global DNA hypo-methylation in *P. sativum* by modulating NAD(P)H and GSH levels in cells (Berglund et al. 2017). In addition, redox components modulate DNA methylation by regulating dicer-like4 (DCL4) and RNAse III-like 1 (RTL1) activities involved in the production of siRNAs likely required for methylation of DNA through the RNA-directed DNA methylation (RdDM) pathway in plants (Charbonnel et al. 2017, Seta et al. 2017). Priming with sulfur and sulfur-containing compounds (GSH and TRXs) has been well documented to play a critical role in plant defense against multiple biotic and abiotic stresses [for details, see Abuelsoud et al. (2016)]. For example, supplementation of GSH and TRXs in *A. thaliana* recovered the DCL4 activity, thus indicating that DCL4 functions under redox regulation (Seta et al. 2017). The DCL4 activation induces siRNA production and promotes DNA methylation (Seta et al. 2017). Furthermore, RTL1 of *Arabidopsis* possesses conserved cysteine (Cys230) in the dsRNA-binding domains that is important for RTL1 cleavage activity. However, GSSG presence leads to glutathionylation of RTL1 at Cys230 residue and inhibits cleavage activity of RTL1 enzyme. However, two glutaredoxin (GRX) members, namely, GRXC1 and GRXC2, can recover glutathionylated RTL1 activity, suggesting redox regulation of RTL1. The siRNA production is negatively regulated by RTL1 prior to DCL-mediated cleavage of the siRNA precursors (Charbonnel et al. 2017). Hence, redox components control generation of siRNA by affecting DCL4 and RTL1 activities and thereby affect DNA methylation in plants.

**Fig. 3:**
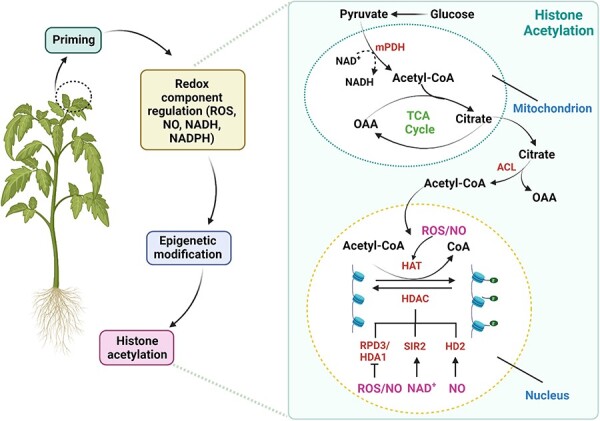
Promotion of histone acetylation as a result of priming through modulation of redox components, which in turn regulates the enzymes involved in the process. In the cytoplasm, glucose undergoes breakdown to pyruvate, which subsequently enters the mitochondria. Within the mitochondria, pyruvate is transformed into acetyl CoA by mPDH, a process involving the reduction of NAD+. The generated acetyl CoA then combines with oxaloacetate (OAA), produced in the tricarboxylic acid cycle, to produce citrate, which exits the mitochondria and enters the cytoplasm. In the cytoplasm, citrate is enzymatically converted back to OAA and acetyl CoA through the action of ATP-citrate lyase (ACL). Acetyl CoA, synthesized in the cytoplasm, subsequently enters the nucleus to serve as the supplier of acetyl groups for the histone acetylation process. HAT uses the acetyl group from acetyl CoA to add acetylation marks (Ac) to the lysine residues of the histone tail. This process weakens the interaction between DNA and histones, facilitating gene expression. HDAC, on the other hand, removes acetyl groups from histones, leading to chromatin compaction. Various HAT and HDAC enzymes are susceptible to the influence of ROS, NO and NAD+. Mitochondrial PDH (mPDH), OAA, ACL, HATs and HDACs reduced potassium dependency 3/histone deacetylase 1 (RDP3/HDA1), silent information regulator 2 (SIR2) and plant-specific histone deacetylase 2 (HDA2), NIC adenine dinucleotide (NAD+). Figure is adapted after modification from RM et al. (2020) available at CCBY4.0. Created using BioRender.

Numerous pieces of evidence indicate that redox components regulate histone acetylation by influencing the accumulation of acetyl CoA, primarily through the modulation of pyruvate dehydrogenase (PDH) activity. (Mews et al. 2017, Sivanand et al. 2018, Sidoli et al. 2019, RM et al. 2020) (**Fig. 3**). The acetyl CoA acts as an acetyl donor for histone acetyltransferases (HATs) (Sidoli et al. 2019). Conversion of pyruvate to acetyl CoA is catalyzed by the PDH complex which uses the cofactor NAD^+^ in its catalytic activity. In *Escherichia coli*, a rise in the ratio of NADH to NAD^+^ inhibits activity of PDH and prevents acetyl CoA formation (Ojima et al. 2012). Similarly, an in vitro study in pea demonstrated that an increase in the NADH*/*NAD^+^ ratio inhibits PDH activity (Miernyk and Randall 1987). Serrano et al. (2019) demonstrated that thermo-priming of *A. thaliana* reprograms metabolic homeostasis to confer heat tolerance, such as by increased levels of acetyl CoA in primed plants compared with unprimed plants, suggesting that heat-priming might result in a decreased NADH*/*NAD^+^ ratio. Moreover, redox components directly regulate activity of HATs and histone deacetylases (HDACs) in plants. For example, Wang et al. (2015a) documented that heat stress induces accumulation of O_2_^−^ that in turn stimulates histone hyperacetylation by directly affecting elevated expression of genes such as *HAT-B* and *General Control Non-depressible 5* (*GCN5*) in maize seedlings. Likewise, *Arabidopsis* mutants, *cat2* and *gr1*, showed elevated levels of H_2_O_2_ accumulation and inability in converting GSSG to GSH, respectively, displaying distinct expression patterns of the GCN5-related acetyltransferase gene (Mhamdi et al. 2010). In *A. thaliana*, pathogen attack inhibits HDA19 activity leading to acetylation and enhanced PR-related gene expression (Choi et al. 2007). Wojtaszek (1997) suggests that ROS regulates HDA19 activity under pathogen attack. For instance, Liu et al. (2015) illustrated that increased production of reactive oxygen species (ROS) causes oxidation of HDA9 and HDA19 in *A. thaliana*, reducing their functionality. This reduction in activity subsequently enhances the histone acetylation of stress-response genes. In plants, the levels of NAD^+^ and the NAD^+^ /NADH ratio impact the activities of sirtuin (SIR2/SIRT-like proteins) HDACs (Hu et al. 2019). Consequently, oxidative stress has the potential to modify the redox state of NAD^+^ and may consequently affect HDAC activity. In line with this suggestion, Zhang et al. (2017) reported that NAD*^+^*-dependent HDACs in rice suppress glycolysis by de-acetylating histones and glyceraldehyde-3-phosphatedehydrogenase. Meanwhile, Mengel et al. (2017) reported that GSH/GSSG reversibly suppresses the activity of HDAC in *A. thaliana*. Hence, priming has been extensively demonstrated to have a major effect on cellular redox homeostasis under various plant stresses, which in turn regulate epigenetic modification as well as transcription of defense-related genes leading to enhanced stress tolerance and a transgenerational effect of priming in plants.

Box 3: Different forms of induced resistance in plants and their regulator defense–signaling pathways

**SAR**: It is a “whole-plant” resistance response developed due to an earlier localized exposure to a pathogen.

**BABA-IR**: BABA is a non-protein amino acid that can induce plant resistance against a wide-spectrum of biotic and abiotic stresses. However, BABA-IR to herbivores has been studied well, particularly its underlying mechanism.

**Rhizobacteria-mediated ISR**: Rhizobacteria-mediated ISR enhances the resistance of uninfected plant parts against a wide range of plant pathogens, similar to the SAR induced by pathogens themselves. In certain instances, rhizobacteria stimulate SAR that relies on SA, while in other cases, they activate an alternative signaling pathway independent of SA.

**VOC-induced resistance**: VOCs are released by some plant growth–promoting organisms and can effectively promote plant systemic resistance against diseases.

**Convergent signaling pathways**: A convergent signaling pathway involves signals from different types of unrelated receptors, and all these signals have a common target.

**JA**: JA is an organic compound that is a member of the jasmonate class of phytohormones. It is biosynthesized from linolenic acid by the octadecanoid pathway. It plays an important role in endogenous signaling and mediates plant defense response against multiple stresses.

**SA**: SA is a phytohormone that plays an important role in plant growth and development, photosynthesis, transpiration, ion uptake and transport. SA mediates plant defense against pathogens through endogenous signaling.

**NPR1**: NPR1 plays a crucial role in triggering both SAR and ISR, serving as a central component in the plant defense signaling network. It acts as a master regulator facilitating communication between the SA and JA/ET responses.

## Chemical and Biological Priming Share Common Signaling and Molecular Pathways as Part of a Complex Network

Studies have demonstrated that chemical and biological priming share some common signaling and molecular pathways for inducing resistance/tolerance against multiple plant stresses (Pastor et al. 2014; for different forms of induced resistance in plants, see **Box 3**). However, this information has been gained only for a limited number of priming agents, and it remains unknown for a vast array of chemical and biological priming agents (Tripathi et al. 2019). Hence, considerable efforts are needed to elucidate the common pathways and elements involved in priming response among different categories of priming agents (Rejeb et al. 2014, Vurukonda et al. 2016, Tripathi et al. 2019). Different priming agents regulate JA, ethylene (ET) and SA-dependent defense response via a direct or indirect effect on common TF genes (Carris et al. 2015). These TF genes include the members belonging to the AP2/EREBP, MYB, MYC, NAC and WRKY families (Hoang et al. 2017). Biological priming agents such as *P. fluorescens*, rhizobacterium, prime *A. thaliana* resistance against *Hyaloperonospora arabidopsidis* by regulating JA- and ET-dependent signaling pathways through induction of TF genes such as *AP2/EREBP* and *MYC2* (Lorenzo et al. 2003, 2004, De Vleesschauwer et al. 2008, Pozo et al. 2008, Van der Ent et al. 2009). Naoumkina et al. (2008) also reported that a yeast-elicitor primes stress response in *Medicago truncatula* through its effect on the expression of TFs of the *AP2/EREBP, MYB* and *WRKY* families. Aamir et al. (2019) documented that *Trichoderma erinaceum* biopriming modulates the WRKY-mediated defense programming against the fungal pathogen *Fusarium oxysporum* in tomato. Chemical priming agents also influence the expression of TF genes, for instance, Samota et al. (2017) reported that priming of seeds with hormonal or chemical elicitors such as SA, methyl jasmonate, and paclobutrazol (PBZ) increased drought tolerance in rice by significantly increasing expression of drought-responsive *RD1* and *RD2* genes belonging to the AP2/ERF family. Furthermore, many genes encoding WRKYs, which regulate the defense response, were induced in hexanoic acid–primed tomato plants (Finiti et al. 2014). Included in the WRKYs induced by hexanoic acid were orthologs of *Arabidopsis* WRKY18, WRKY33, WRKY40, WRKY53, and WRKY75, all of which play a role in the defense response in Arabidopsis, especially against *B. cinerea* (AbuQamar et al. 2006, Pandey et al. 2010, Birkenbihl et al. 2012). Similarly, it has been revealed that OsWRKY*45* plays a crucial role in benzothiadiazole (BTH)-induced defense responses against blast disease in rice (Shimono et al. 2012). Apart from BTH, alternative chemical priming agents like PBZ and Tiadinil have been shown to trigger resistance against blast disease in rice, partially through a pathway dependent on WRKY45 (Shimono et al. 2012). Microarray analysis has identified WRKY45-regulated genes responsive to BTH (Nakayama et al. 2013). Additionally, isotianil treatment induces the expression of certain defense-related genes, such as *OsWRKY45*, involved in SA signaling to initiate a defense response against biotrophic pathogens (Toquin et al. 2012).

Interestingly, priming with β-amino-butyric acid (BABA, chemical agent) and rhizobacteria (biological agent) against pathogenic fungi and oomycetes has been observed to be associated with the common mechanism of formation of callose-rich papillae (Kishimoto et al. 2005, Ton et al. 2005). For example, rhizobacteria such as *P. fluorescens* and BABA stimulate disease resistance in *Arabidopsis* by priming for increased deposition of callose-rich papillae following infection by the oomycete *Hyaloperonospora arabidopsidis* (Van der Ent et al. 2009). This priming process is governed by convergent pathways that rely on phosphoinositide- and ABA-dependent signaling components (Ton et al. 2009, Van der Ent et al. 2009). In both scenarios, impaired priming was observed in *ibs2* mutants (deficient in polyphosphoinositide phosphatase) and *ibs3* mutants (defective in the ABA biosynthetic enzyme zeaxanthin epoxidase), suggesting that rhizobacteria-induced systemic resistance (ISR) and BABA-induced resistance (IR) against *H. arabidopsidis* share similar phosphoinositide- and ABA-dependent signaling components (Ton et al. 2005, Van der Ent et al. 2009). Similarly, rhizobacterium- and chemical-mediated priming for disease resistance in plants were found to be dependent on ET, JA and non-expresser of PR-1 (NPR1) signaling. For example, rhizobacterium (e.g. *P. putida* (LSW17S)) and chemical agents (e.g. benzo(1,2,3)thiadiazole-7-carbothioic acid *S*-methyl ester (BTH) and (*R*)‐β‐homoserine) priming induce disease resistance in *Arabidopsis*, by regulating common ET, JA and NPR1 signaling pathways (Kohler et al. 2002). Studies have reported that priming with IAA and rhizobacteria (producing IAA-producing and ACC deaminase) enhances stress tolerance by sharing common elements i.e. modulation of endogenous plant hormone concentration (ABA, JA and ET) and genes (*DREB, MYB, WRKY*, *bZIP*, etc.) (Chang et al. 2014, Palaniyandi et al. 2015). Moreover, priming with *Azospirillum brasilense* and NO for tolerance against abiotic stresses in plants involves the same IAA pathway (Molina-Favero et al. 2008). Under aerobic conditions, it has been noted that *A. brasilense* generates significant amounts of No, which subsequently serves as a signaling molecule within an IAA-induced pathway that regulates the development of adventitious roots. (Molina-Favero et al. 2008, Liu et al. 2019).

From the above, it is clear that various biological and chemical priming agents share common signaling and molecular elements. However, this information is available for only a few priming agents belonging to both categories. Hence, extensive research efforts are needed to elucidate detailed information about the common elements and pathways involved in biological and chemical priming, employing state-of-the-art omics platforms and modern gene editing approaches such as CRISPR/Cas.

Box 4: Questions under investigation

Do all plants possess the competence to be primed? Is this competence tissue—or organ—specific?

Does priming of a plant get influenced by the developmental stage of plants and surrounding environmental conditions?

What are the standard conditions that have to be maintained for obtaining maximum benefits of priming?

How do the signaling mechanisms involved in stress priming operate and what are their associated molecular, physiological and ecological facets in order to comprehensively understand the influence of the constantly fluctuating environment on the memory of a plant regarding stresses?

Is priming able to affect the reproductive potential of the plant besides enhancing their growth and tolerance against stresses?

## Future Directions and Unanswered Questions

Priming enables the plants to remember and recall the experiences generated in response to first exposure to stress or other stimuli, especially at the molecular level, and to adapt accordingly when the biotic and abiotic stresses are encountered in future. However, the molecular mechanisms underlying priming still require detailed elucidation, so as to establish priming as an effective strategy toward sustainable agriculture (for key questions under investigation, see **Box 4**). It will also help in development of crops with improved resistance against stresses along with enhanced productivity. However, the differences observed between laboratory and field experiments represent a major challenge for large-scale implementation of priming in plants. Plants in the field are generally exposed to multiple stresses simultaneously. In recent years, studies have been carried out to understand plant responses to more than one stress factor acting concurrently (Peck and Mittler 2020, Zandalinas et al. 2021). Further research in this context will assist in analyzing and predicting plant responses under such realistic conditions. Among various prevalent priming strategies, chemical priming has received major attention lately in inducing tolerance against different abiotic stresses, either acting solely or in combination. Another aspect that needs investigation is the inter-relation between priming and subsequent stress memory generation in order to highlight the importance of stress priming in plants. Employing contemporary genetic approaches and system analysis in future research will substantiate our knowledge on the spatial and temporal particulars of the mechanisms incorporated in the priming of plants.

## Data Availability

No new datasets were generated or analyzed in this study.
